# Gender differences in frailty transition and its prediction in community-dwelling old adults

**DOI:** 10.1038/s41598-022-11358-7

**Published:** 2022-05-05

**Authors:** Nina Mielke, Alice Schneider, Dörte Huscher, Natalie Ebert, Elke Schaeffner

**Affiliations:** 1 Institute of Public Health, Charité – Universitätsmedizin Berlin, corporate member of Freie Universität Berlin and Humboldt-Universität zu Berlin, Charitéplatz 1, 10117 Berlin, Germany; 2 Institute of Biometry and Clinical Epidemiology, Charité – Universitätsmedizin Berlin, corporate member of Freie Universität Berlin and Humboldt-Universität zu Berlin, Charitéplatz 1, 10117 Berlin, Germany

**Keywords:** Geriatrics, Epidemiology

## Abstract

Frailty is very common in old age and often associated with adverse events. Transitioning between frailty states is possible in both directions (improvement and worsening) offering targets for interventions. Frailty is more prevalent in women, but little is known about the impact of gender on frailty transition. The aim of this study is to identify gender differences for frailty transition in older adults and to develop gender-stratified prognostic prediction models for frailty transition. We performed a longitudinal analyses of the Berlin Initiative (cohort) Study with a frailty follow-up of 2.1 years. Description of frailty transition using the frailty phenotype and development of prognostic prediction models using multivariable logistic regressions for transition (improvement or worsening) stratified by gender following the TRIPOD statement were performed. In total, the study population consisted of 1158 community-dwelling adults with a mean age of 84.4 years and of whom 55% were women. Out of 1158 participants 225 (19%) were robust, 532 (46%) prefrail and 401 (35%) frail. After 2.1 (IQR 2.0–2.3) years, half of the participants had transitioned between frailty states. Men worsened more often and those who were already frail died more often than women. Gender-stratified prediction models for frailty transition demonstrated that some predictors (age, self-rated health, cognitive impairment, baseline frailty status) were included in all models. While stroke, diabetes mellitus, smoking and glomerular filtration rate were unique predictors in the models for females, osteoarthritis, hospitalization and education were predictors in the models for males. There are gender differences in frailty transition rates, patterns and prediction. This supports the importance of considering gender when addressing frailty and targeting interventions in old age.

## Introduction

Frailty has been described as one of the greatest challenges facing the aging populations^1^. It is described as a biological syndrome with accelerated decrease in physiological reserve and resistance to stressors^2,3^. Individuals degrade across multiple physiological systems and these cumulative declines result in an increased susceptibility to adverse events^2^. These adverse events include an increased risk of falls, hospitalization, disability in activities of daily living, need of nursing home, or mortality^2,4^. Among several different instruments to assess frailty one of the most frequently used is the frailty phenotype by Fried^5,6^. Using this instrument in populations 65 years and older the prevalence of frailty ranged between 4.0% and 17.0%^6^. With older age the prevalence of frailty increases further^6,7^. Overall, prevalence and incidence of frailty are higher in women than in men^8,9^.

Frailty is also a dynamic process and its status can continuously change by worsening or even improving over time^10,11^. Understanding the transition of frailty especially in very old age is important since it potentially offers timely approaches for interventions. A recent systematic review and meta-analysis showed that the frailty status of community-dwelling older adults worsened in about 30% within a mean follow-up of 3.9 years, whereas it improved in 13%^12^. To define specific targets for intervention, the factors associated with frailty transition have to be identified. Since frailty is more common in women, frailty transition—concerning its pattern and risk factors—could also be different for men and women. So far, most studies did not stratify by gender and identified older age, hospitalization, previous stroke, cognitive impairment, previous cancer, osteoarthritis, diabetes, socio economic status, COPD, congestive heart failure, polypharmacy, urinary incontinence or frailty baseline status to be associated with frailty worsening regardless of gender^11,13–18^.

Apparently, there is a lack of evidence regarding the gender impact on frailty transition^19^ and the little data available in old age are inconsistent^12^. Thus, the aim of this study was to analyze gender differences of frailty transition in a population of community-dwelling older adults by (1) describing the different transition patterns of improving, no change or worsening by gender and (2) to develop prognostic prediction models for frailty transition stratified by gender following the TRIPOD guidelines^20^.

## Methods

The focus of this work was the analysis of frailty transition with a special emphasis on gender differences using a longitudinal approach with data from the Berlin Initiative Study (BIS).

### Study population

**Figure 1:**
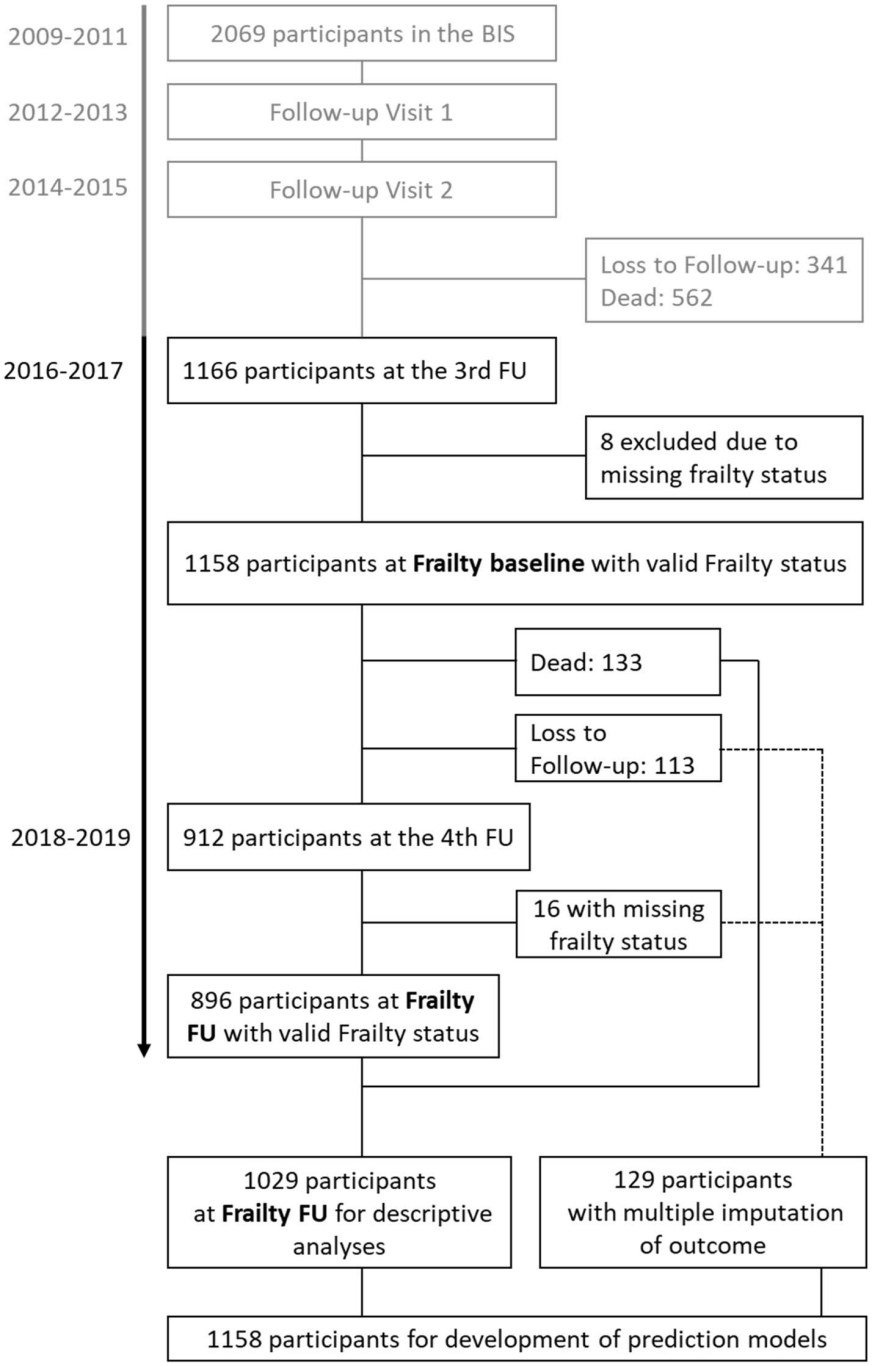
Overview of the Berlin Initiative Study (BIS) population. The flowchart shows the composition of the study population and which participants were available for certain analyses. The light gray section of the figure indicates the part of the BIS study that preceded this study. The frailty assessment was implemented at the 3rd follow-up (FU) of the BIS, defining the baseline visit for this study.

The BIS is a population-based cohort study of older adults initially enrolling 2069 participants between November 2009 and July 2011 aged 70 years and older. In order to be eligible, participants were not allowed to be dialysis patients, kidney transplant recipients or nursing home residents and had to be members of the AOK [“Allgemeine Ortskrankenkasse” (AOK)-Nordost—Berlin’s largest statutory health insurance fund]. Prior to the study, written informed consent was obtained from all participants. Participants were revisited every two years and those who were, e.g. for physical reasons, not able to come to a study site were visited at home. The study was approved by the ethics committee, Charité, Berlin, Germany (EA2/009/08). The study concept and design of the BIS have been described in detail^21,22^. The frailty questionnaire was implemented starting with the third follow-up BIS visit. For this analysis, we used data from the third and fourth BIS follow-up and will refer to these as baseline (3rd FU: 2016–2017) and follow-up (4th FU: 2018–2019), respectively (Fig. 1). The baseline study population consisted of 1166 participants. We excluded eight participants with missing data in the frailty score. The final study population consisted of 1158 eligible participants (Fig. 1). Closure of the data set was October 31st 2019.

### Frailty assessment

Frailty status was determined by a modified Frailty score according to Fried^2^. Frailty was defined as having at least three of the following five items: shrinking, exhaustion, low physical activity, slowness and weakness. Participants with one or two of the five items were defined as prefrail, and participants with none of the five items were defined as robust. Further details are described in Supplement A.

### Frailty transition

Primary outcome was the frailty transition state at follow-up (median follow up time 2.1 years). Frailty transition was defined as improvement (frail to prefrail, frail to robust, prefrail to robust), worsening (robust to prefrail, robust to frail, prefrail to frail, any status to death) or no change (staying robust, prefrail, or frail).

### Co-variables assessment

At each study visit, extensive data on demographics, lifestyle variables, morbidity, medication, anthropometric and geriatric assessment were conducted using a standardized computer-based questionnaire. Additionally, blood and urine samples were taken for instant analysis. Laboratory processing and measurement have been described previously^22,23^. In addition, the study data were complemented with AOK claims data. By this unique approach, self-reported data on e.g. hospitalizations and morbidities could be supplemented with verified claims data (i.e. diagnoses coded by the 10h Revision of the International Statistical Classification of Diseases and Related Health Problems (ICD-10)), and information from participants who were no longer seen at follow up was available. Also, the exact date of death of all participants who had died during follow-up was ascertained with the claims data. The following covariates were derived from BIS data: age, sex, the short version of the CASMIN classification of education^24^, living alone (yes, no), cognitive impairment (yes, no)^25^, smoking status (never, ever), self-rated health in two categories (excellent/good vs. moderate/poor/very poor) and body mass index (< 24.9, 25–29.9 or ≥ 30 kg/m^2^). Information on glomerular filtration rate (GFR, < 60 or ≥ 60 ml/min per 1.73m^2^) estimated by the BIS2 Equation^23^ and albuminuria (albumin-to-creatinine ratio ≥ 30 mg/g) as well as polypharmacy (≥ 5 medications) and diabetes mellitus (hemoglobin A1c ≥ 6.5% or intake of any antidiabetic drug) were also derived from the BIS data. The Charlson Comorbidity Index (CCI)^26^, osteoarthritis (ICD-10: M15-M19), COPD (ICD-10: J41-J44) and congestive heart failure (ICD-10: I50, I11.0, I13.0, I13.2, I25) were ascertained by a combination of self-reported BIS data validated by ICD-10 coded medical reports and AOK claims data as were stroke (ICD-10: I61, I63, I64), cancer (ICD-10: C00-C97 except C44) and the number of all-cause hospitalizations two years prior to baseline.

### Statistical analysis

The baseline characteristics of the study population as well as the transition states were described for the total population and stratified by gender. Descriptive analysis included absolute and relative frequencies for categorical variables, for continuous variables either means with standard deviations or medians with interquartile range.

To develop prognostic prediction models for the transition between frailty states (worsening or improvement) after a median duration of 2.1 years we used multivariable logistic regressions. It was not possible to develop prognostic models for no change. Candidate predictors were selected based on medical knowledge (Table 1). The number of candidate predictors was restricted by the number of events^20,27^. Multicollinearity was assessed using the variance inflation factor^28^. Prediction models for the two transition outcomes were also stratified by gender, resulting in 4 models in total. Multiple imputation by chained equation was used to address all missing values (Suppl. Table 2). All predictor candidates and frailty status at follow up were used in the multiple imputations to generate 10 imputed data sets. For variable selection, we combined multiple imputation and bootstrapping. From every imputed data set we drew 200 bootstrap samples and applied automatic backward selection based on AIC. A variable was finally selected, if the selection frequency was ≥ 50% of the 2000 samples^29^. The selected predictor sets were finally tested in the original imputed data; pooled results are reported. For model discrimination, the optimism-corrected AUC was estimated as a measurement of the concordance index (c-index)^30^. The c-index is the probability that a participant with a certain change in frailty status will be assigned in the model with a higher probability in experiencing that outcome than a participant who did not experience the outcome. The standard error of AUC for the estimation of the 95% confidence interval was estimated by 2000 bootstrap replications. To calculate the bias-corrected model calibration we drew 200 bootstrap samples from every imputed data set^31^. To illustrate the model calibration we plotted the observed probabilities of all 10 imputed data sets against the predicted probabilities.

**Table 1:** Main baseline characteristics by gender.

	Total	Women	Men
n (%)	1158 (100)	637 (55)	521 (45)
Age in years, mean (SD)	84.4 (5.6)	84.1 (5.6)	84.8 (5.7)
Education (CASMIN-short), n (%)			
Low	686 (59)	417 (66)	269 (52)
Middle	235 (20)	148 (23)	87 (17)
High	232 (20)	71 (11)	161 (31)
Unknown	5 (0.4)	1 (0.2)	4 (0.8)
Smoking, n (%)			
Never	622 (54)	458 (72)	164 (32)
Ever	532 (46)	176 (28)	356 (68)
Unknown	4 (0.3)	3 (0.5)	1 (0.2)
Self-rated health, n (%)			
Excellent / good	521 (45)	267 (42)	254 (49)
Moderate / poor / very poor	634 (55)	368 (58)	266 (51)
Unknown	3 (0.3)	2 (0.3)	1 (0.2)
Living alone, n (%)	552 (48)	393 (62)	159 (31)
Unknown	38 (3)	25 (4)	13 (2)
BMI in kg/m^2^, n (%)			
< 25	377 (33)	225 (35)	152 (29)
25–29.9	509 (44)	251 (39)	258 (50)
≥ 30	255 (22)	154 (24)	101 (19)
Unknown	17 (1)	7 (1)	10 (2)
Polypharmacy, n (%)	848 (73)	470 (74)	378 (73)
Unknown	1 (0.1)	0 (0)	1 (0.2)
Hospitalization, n (%)			
0	489 (42)	284 (45)	205 (39)
1–2	448 (39)	230 (36)	218 (42)
≥ 3	221 (19)	123 (19)	98 (19)
CCI, median (IQR)	10 (7–12)	9 (7–12)	10 (8–13)
Cognitive impairment, n (%)	95 (8)	55 (9)	40 (8)
Unknown	68 (6)	32 (5)	36 (7)
Hypertension, n (%)	958 (83)	533 (84)	425 (82)
Unknown	2 (0.2)	1 (0.2)	1 (0.2)
Stroke, n (%)	156 (14)	75 (12)	81 (16)
Congestive Heart failure, n (%)	756 (65)	390 (61)	366 (70)
Diabetes mellitus, n (%)	341 (29)	171 (27)	170 (33)
Unknown	15 (1)	8 (1)	7 (1)
Cancer, n (%)	315 (27)	134 (21)	181 (35)
Osteoarthritis, n (%)	935 (81)	558 (88)	377 (72)
COPD, n (%)	450 (39)	254 (40)	196 (38)
eGFR_BIS2_ < 60 ml/min/1.73m^2^, n (%)	847 (73)	468 (74)	379 (73)
Unknown	34 (3)	22 (3)	12 (2)
ACR ≥ 30 mg/g, n (%)	306 (26)	137 (22)	169 (32)
Unknown	77 (7)	49 (8)	28 (5)
Frailty status, n (%)			
Robust	225 (19)	115 (18)	110 (21)
Prefrail	532 (46)	282 (44)	250 (48)
Frail	401 (35)	240 (38)	161 (31)
Frailty transition after 2.1 years, n (%)			
Improvement	160 (14)	101 (16)	59 (11)
No change	547 (47)	304 (48)	243 (47)
Worsening	322 (28)	162 (25)	160 (31)
Unknown	129 (11)	70 (11)	59 (11)

*SD* standard deviation, *BMI* body mass index, *CCI* Charlson Comorbidity Index, *COPD* chronic obstructive lung disease, *eGFR* estimated glomerular filtration rate, *ACR* albumin to creatinine ratio.

All statistical analyses were conducted with IBM SPSS Statistics (Version 25.0; IBM Corp, Armonk, NY), or R (Version 3.6.1; R Foundation for Statistical Computing, Vienna, Austria).

### Ethical approval

The study was approved by the ethics committee, Charité—Universitätsmedizin Berlin, Germany (EA2/009/08) and is in accordance with the 1964 Helsinki declaration and its later amendments. Written informed consent was obtained from all individual participants included in the study.

## Results

### Characteristics of the study sample

The main baseline characteristics of the 1158 study participants stratified by gender are displayed in Table 1. The mean age (SD) was 84.4 (5.6) years and 55% were women. About 60% had received a low education, half of the participants were living alone, and 45% rated their health as excellent or good. In the two years prior to baseline 58% of the participants had been hospitalized, 14% had experienced a stroke and 27% suffered from cancer. At baseline 8% were cognitively impaired, 29% had diabetes mellitus, and about three quarters had an impaired kidney function (GFR_BIS2_ < 60 ml/min/1.73 m^2^) or took ≥ 5 medications; with regard to frailty 19% were robust and 35% frail.

Women had received a lower education more often than men (66% vs. 52%), were more often never-smokers (72% vs. 32%) or living alone (62% vs. 31%), and exhibited less morbidities such as stroke (12% vs. 16%), heart failure (61% vs. 70%) and cancer (21% vs. 35%), but were diagnosed more often with osteoarthritis (88% vs. 72%) than men.

### Frailty transition

**Figure 2:**
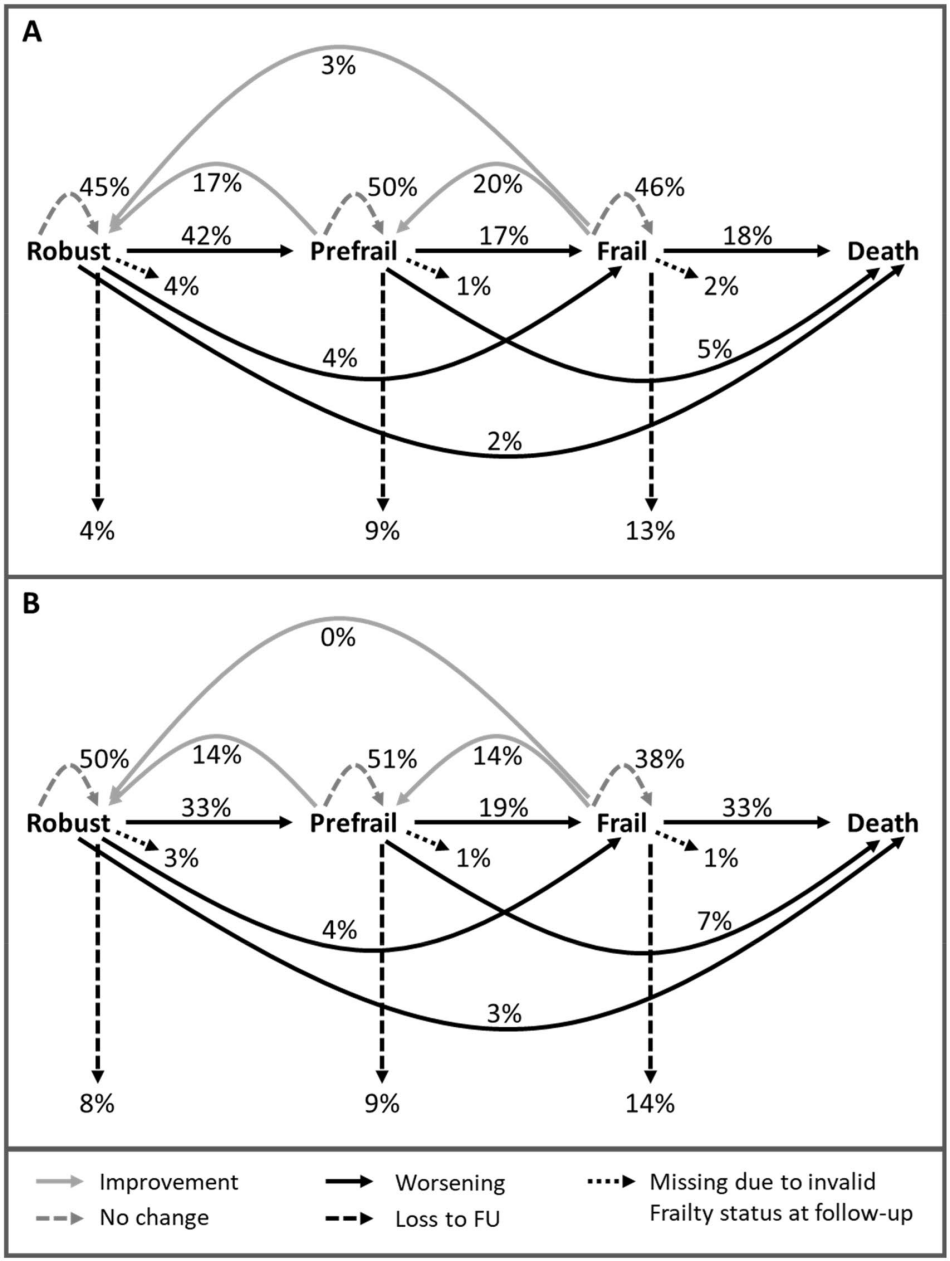
Transitions between frailty states and death after 2.1 years (**A**) Women (N = 637): at baseline 115 (18%) were robust, 282 (44%) prefrail and 240 (38%) frail. (**B**) Men (N = 521): at baseline 110 (21%) were robust, 250 (48%) prefrail and 161 (31%) frail. The relative frequencies of the frailty status transition are displayed by gender and refer to the baseline status. In total, all frequencies add up to 300%.

After 2.1 (2.0–2.3) years, frailty transition status could be determined for 1029 of the initial 1158 participants (Fig. 1). Out of 1029 participants 482 (42%) had transitioned between frailty states or death, with improvement in 160 (14%) and worsening in 322 (28%) participants (Table 1). Of those who worsened 133 (41%) had died (Supplement Table 1). Participants who were already frail at baseline, died more often (24%) compared to prefrail (6%) or robust (2%) participants. Transitions between adjacent frailty states were more frequent than those across several frailty states (Fig. 2). Overall, women improved slightly more often than men, while men worsened more often. More women than men who were frail remained frail (46% vs. 38%) and men who were frail died more frequently (33% men vs. 18% women).

### Baseline characteristics by frailty transition and stratified by gender

Participants who worsened were older, self-rated their health more often as moderate or (very) poor, were more often cognitively impaired, demonstrated more often a decreased kidney function (GFR < 60 ml/min/1.73m^2^) or kidney damage (ACR ≥ 30 mg/g) and had a higher prevalence of morbidities such as stroke or heart failure compared to those who improved or remained in their frailty status (Table 2).

**Table 2:** Main baseline characteristics by frailty transition status after 2.1 years and stratified by gender.

	Improvement			No Change			Worsening		
	Women	Men	Total	Women	Men	Total	Women	Men	Total
n	101	59	160	304	243	547	162	160	322
Age in year, mean (SD)	82.9 (5.1)	83.4 (5.2)	83.1 (5.1)	83.7 (5.4)	84.3 (5.5)	84.0 (5.4)	85.0 (6.1)	86.0 (6.0)	85.5 (6.1)
Education (CASMIN-short), n (%)									
Low	69 (68)	26 (44)	95 (59)	197 (65)	124 (51)	321 (59)	104 (64)	88 (55)	192 (60)
Middle	22 (22)	16 (27)	38 (24)	76 (25)	41 (17)	117 (21)	36 (22)	22 (14)	58 (18)
High	9 (9)	17 (29)	26 (16)	31 (10)	78 (32)	109 (20)	22 (14)	47 (29)	69 (21)
Unknown	1 (1)	0 (0)	1 (0.6)	0 (0)	0 (0)	0 (0)	0 (0)	3 (2)	3 (0.9)
Smoking, n (%)									
Never	73 (72)	17 (29)	90 (56)	223 (73)	81 (33)	304 (56)	106 (65)	50 (31)	156 (48)
Ever	28 (28)	42 (71)	70 (44)	80 (26)	162 (67)	242 (44)	54 (33)	110 (69)	164 (51)
Unknown	0 (0)	0 (0)	0 (0)	1 (0.3)	0 (0)	1 (0.2)	2 (1)	0 (0)	2 (0.6)
Self-rated health, n (%)									
Excellent / good	46 (46)	31 (53)	77 (48)	129 (42)	131 (54)	260 (48)	61 (38)	68 (43)	129 (40)
Moderate /poor / very poor	55 (55)	28 (48)	83 (52)	173 (57)	111 (46)	284 (52)	101 (62)	92 (58)	193 (60)
Unknown	0 (0)	0 (0)	0 (0)	2 (0.7)	1 (0.4)	3 (0.5)	0 (0)	0 (0)	0 (0)
Living alone, n (%)	56 (55)	16 (27)	72 (45)	180 (59)	77 (32)	257 (47)	113 (70)	46 (29)	159 (49)
Unknown	3 (3)	2 (3)	5 (3)	12 (4)	5 (2)	17 (3)	7 (4)	5 (3)	12 (4)
BMI in kg/m^2^, n (%)									
< 25	32 (32)	15 (25)	47 (29)	107 (35)	62 (26)	169 (31)	61 (38)	53 (33)	114 (35)
25–29.9	39 (39)	32 (54)	71 (44)	122 (40)	124 (51)	246 (45)	64 (40)	72 (45)	136 (42)
≥ 30	28 (28)	12 (20)	40 (25)	74 (24)	52 (21)	126 (23)	36 (22)	32 (20)	68 (21)
Unknown	2 (2)	0 (0)	2 (1)	1 (0.3)	5 (2)	6 (1)	1 (0.6)	3 (2)	4 (1)
Polypharmacy, n (%)	73 (72)	41 (70)	114 (71)	223 (73)	174 (72)	397 (73)	125 (77)	121 (76)	246 (76)
Hospitalization, n (%)									
0	45 (45)	28 (48)	73 (46)	141 (46)	104 (43)	245 (45)	67 (41)	48 (30)	115 (36)
1–2	36 (36)	20 (34)	56 (35)	104 (34)	98 (40)	202 (37)	61 (38)	72 (45)	133 (41)
≥ 3	20 (20)	11 (19)	31 (19)	59 (19)	41 (17)	100 (18)	34 (21)	40 (25)	74 (23)
CCI, median (IQR)	8 (6–11)	10 (8–12)	9 (7–11)	9 (7–12)	10 (7–13)	9 (7–12)	10 (7–13)	12 (9–14)	11 (8–13)
Cognitive impairment, n (%)	2 (2)	1 (2)	3 (2)	22 (7)	12 (5)	34 (6)	22 (14)	16 (10)	38 (12)
Unknown	3 (3)	1 (2)	4 (3)	10 (3)	10 (4)	20 (4)	11 (7)	17 (11)	28 (9)
Hypertension, n (%)	86 (85)	49 (83)	135 (84)	253 (83)	199 (82)	452 (83)	139 (86)	133 (83)	272 (85)
Unknown	0 (0)	0 (0)	0 (0)	0 (0)	0 (0)	0 (0)	1 (0.6)	0 (0)	1 (0.3)
Stroke, n (%)	7 (7)	9 (15)	16 (10)	35 (12)	27 (11)	62 (11)	26 (16)	31 (19)	57 (18)
Congestive Heart failure, n (%)	45 (45)	40 (68)	85 (53)	186 (61)	161 (66)	347 (63)	112 (69)	127 (79)	239 (74)
Diabetes mellitus, n (%)	18 (18)	15 (25)	33 (21)	82 (27)	79 (33)	161 (29)	50 (31)	56 (35)	106 (33)
Unknown	0 (0)	0 (0)	0 (0)	0 (0)	0 (0)	0 (0)	4 (3)	2 (1)	6 (2)
Cancer, n (%)	16 (16)	27 (46)	43 (27)	62 (20)	75 (31)	137 (25)	35 (22)	61 (38)	96 (30)
Osteoarthritis, n (%)	92 (91)	43 (73)	135 (84)	267 (88)	168 (69)	435 (80)	142 (88)	127 (79)	269 (84)
COPD, n (%)	35 (35)	21 (36)	56 (35)	121 (40)	87 (36)	208 (38)	67 (41)	60 (38)	127 (39)
eGFR_BIS2_ < 60									
ml/min/1.73m^2^, n (%)	68 (67)	43 (73)	111 (69)	218 (72)	170 (70)	388 (71)	128 (79)	127 (79)	255 (79)
Unknown	4 (4)	0 (0)	4 (3)	6 (2)	2 (0.8)	8 (2)	5 (3)	4 (3)	9 (3)
ACR ≥ 30 mg/g, n (%)	15 (15)	19 (32)	34 (21)	57 (19)	69 (28)	126 (23)	50 (31)	65 (41)	115 (36)
Unknown	7 (7)	1 (2)	8 (5)	22 (7)	10 (4)	32 (6)	11 (7)	10 (6)	21 (7)
Frailty status, n (%)									
Robust	n.a	n.a	n.a	52 (17)	55 (23)	107 (20)	55 (34)	43 (27)	98 (30)
Prefrail	48 (48)	36 (61)	84 (53)	142 (47)	127 (52)	269 (49)	64 (40)	64 (40)	128 (40)
Frail	53 (53)	23 (39)	76 (48)	110 (36)	61 (25)	171 (31)	43 (27)	53 (33)	96 (30)

*SD* standard deviation, *n.a.* not applicable, *BMI* body mass index, *CCI* Charlson Comorbidity Index, *COPD* chronic obstructive lung disease, *eGFR* estimated glomerular filtration rate, *ACR* albumin to creatinine ratio.

While men who worsened were more likely to have received a lower education compared to those who improved their frailty status (55% vs. 44%), education did not differ between transition groups in women (64% vs. 68%). About one third of men were living alone independent of frailty transition. In women who worsened 70% were living alone compared to 59% who remained or 55% of those who improved in their frailty status. Prior hospitalizations for women did not differ between the frailty transition outcomes. However, men who worsened were more often previously hospitalized (70%) compared to men who maintained (57%) or improved (52%) their frailty status (Table 2).

### Prediction of improvement

An improvement of the frailty status could be observed in 116 out of 522 women (22%) and in 70 out of 411 men (17%) after multiple imputation of all missing values (Table 3, Supplement Table 2 and 3). Out of the 18 candidate predictor variables considered, after the bootstrap selection process for the logistic regression models eight variables remained for women and seven for men. Both gender-specific models included the predictors age, self-rated health, cognitive impairment, frailty baseline status, and cancer. For women, the model additionally included heart failure (OR: 0.41; 95%-CI: 0.25–0.68), stroke (OR: 0.46; 95%-CI: 0.19–1.14), but not statistically significant when tested in the original imputed data set, and diabetes mellitus (OR: 0.50; 95%-CI: 0.27–0.90) with reduced odds between 50 and 59% for improvement if one had these comorbidities. The c-index (95%-CI) for discrimination was 0.74 (0.72–0.76). The calibration intercept ranged from -0.16 to -0.05 and the calibration slope from 0.84 – 0.90 (Supplement Fig. 1A).

**Table 3:** Prognostic prediction models for frailty improvement or worsening after 2.1 years in community-dwelling older adults stratified for gender.

	Improvement		Worsening	
	Women	Men	Women	Men
N	522	411	637	521
Number of events	116	70	174	173

Predictor Candidates*	Odds Ratio (95% CI)	Odds Ratio (95% CI)	Odds Ratio (95% CI)	Odds Ratio (95% CI)
Age (1 year)	0.93 (0.88–0.98)	0.87 (0.82–0.93)	1.06 (1.01–1.12)	1.09 (1.05–1.14)
Self-rated health, moderate-very poor	0.56 (0.32–0.97)	0.42 (0.22–0.82)	1.95 (1.24–3.07)	1.67 (1.07–2.62)
Cognitive impairment	0.14 (0.03–0.73)	0.18 (0.02–1.40)	3.70 (1.73–7.92)	2.43 (1.20–4.93)
Frailty status at baseline				
Robust	n.a	n.a	30.11 (13.73–66.06)	8.03 (3.68–17.51)
Prefrail	0.23 (0.13–0.41)	0.29 (0.15–0.57)	4.57 (2.53–8.28)	1.95 (1.14–3.35)
Frail	Ref	Ref	Ref	Ref
Congestive Heart failure	0.41 (0.25–0.68)	–	1.60 (1.00–2.57)	1.63 (0.98–2.72)
Stroke	0.46 (0.19–1.14)	–	1.65 (0.90–3.02)	–
Diabetes mellitus	0.50 (0.27–0.90)	–	1.43 (0.90–2.27)	
Cancer	0.72 (0.38–1.36)	1.65 (0.91–3.01)	–	–
Living alone	–	0.63 (0.33–1.21)	1.57 (0.95–2.58)	–
Education (CASMIN-short)				
Low	–	Ref	–	–
Middle	–	1.98 (0.96–4.10)	–	–
High	–	1.15 (0.58–2.31)	–	–
ACR ≥ 30 mg/g	–	–	1.74 (1.05–2.89)	1.85 (1.12–3.06)
Smoking	–	–	1.59 (1.01–2.50)	–
eGFR_BIS2_ < 60 ml/min/1.73 m^2^	–	–	1.59 (0.90–2.79)	–
Osteoarthritis	–	–	–	1.44 (0.90–2.31)
Hospitalization				
0	–	–	–	Ref
1–2	–	–	–	1.36 (0.83–2.25)
≥ 3	–	–	–	1.72 (0.96–3.08)
Model discrimination				
c-Index	0.74 (0.72–0.76)	0.68 (0.66–0.70)	0.76 (0.75–0.77)	0.66 (0.65–0.67)

*Predictor Candidates not included in any model: BMI, Polypharmacy, COPD.

For men, the model additionally included living alone (OR: 0.63; 95%-CI: 0.33–1.21) and education. The c-index (95%-CI) of this logistic model was 0.68 (0.66–0.70). The calibration intercept ranged from − 0.32 to − 0.21 and the calibration slope from 0.77 to 0.84 (Supplement Fig. 1B).

### Prediction of worsening

A worsening of the frailty status could be observed in 174 of 637 (27%) women and in 173 of 521 (33%) men after multiple imputation of all missing values (Table 3, Supplement Table 2 and 3). Out of the 18 candidate predictor variables considered, after the bootstrap selection process for the logistic regression models 11 remained for women and 8 for men (Table 3). Both gender-specific prediction models included age, self-rated health, heart failure, kidney damage (ACR), cognitive impairment (women: OR 3.70, 95%-CI: 1.73–7.92; men: OR 2.43, 95%-CI: 1.20–4.93) and frailty baseline status, the latter two being the variables with the highest odds for worsening. The model for women additionally included stroke, diabetes mellitus, living alone, smoking and kidney function (GFR). The c-index (95%-CI) for discrimination was 0.76 (0.75–0.77). The calibration intercept ranged from -0.09 to -0.07 and the calibration slope from 0.88 to 0.90 (Supplement Fig. 1C). The model for men additionally included osteoarthritis and hospitalization. The c-index (95%-CI) of this logistic model was 0.66 (0.65–0.67). The calibration intercept ranged from − 0.09 to − 0.07 and the calibration slope from 0.84 to 0.88 (Supplement Fig. 1D).

## Discussion

In this study, we were able to demonstrate that over the course of 2.1 years, half of the participants transitioned into a different frailty status and of those two thirds were worsening. Men worsened more often than women, and if already frail at baseline men died more often than women. Prediction models for frailty improvement or worsening stratified by gender included age, self-rated health, cognitive impairment, heart failure, living alone, ACR, cancer and baseline frailty as common predictors for both genders. Osteoarthritis, hospitalization, and education however, were unique to men, whereas stroke, diabetes, smoking or eGFR were unique to women.

To our knowledge, this is the first study to report gender differences in frailty transition among German older adults. Studies on frailty have been conducted in a variety of settings and have shown that the prevalence of frailty is higher in women than in men^6^ consistent with our results. Our focus was on frailty transition over time. A recent meta-analysis showed that about half of the older adults (mean age between 64 and 79 years) maintain their frailty status over about four years with no significant gender differences in transition rates^12^. In contrast to their findings, we observed more improvement in women but more worsening in men, with identical rates of no change. Reason for this may lie in the high degree of heterogeneity within the studies included in the gender-specific meta-analysis^12^. The overall observation length was longer, participants were younger and by pooling the data from 16 studies they had a bigger sample size.

Studies investigating gender-specific frailty transition are scarce. The few studies investigating frailty transition by gender often only reported the transition rates^18,32,33^. We characterized older adults by their transition pattern and thereby identified gender differences for education, living alone, BMI, the number of hospitalizations, cancer and osteoarthritis. Furthermore, we developed gender-specific prognostic prediction models following the TRIPOD guidelines^20^ to identify predictors for frailty transition. We included the above variables and factors identified in studies mostly not stratifying by gender^11,13,15–18,33–36^. The prediction models for females included more variables and had a higher discrimination ability compared to the models in men that only demonstrated possibly helpful discrimination ability. These findings are in concordance with a study by Lee et al. (2014), where also more variables were included in the models for females, suggesting gender-specific predictors in men have not all yet been identified^13^. However the study did not report discrimination measures. With respect to model calibration, all four prediction models were well calibrated, those for women slightly better than those for men. We acknowledge that our models need to be validated externally before deriving clinical implications^37,38^.

The prediction models could help to identify older adults likely to worsen within the following two years to initiate preventive interventions or to improve the basis for shared decision making. So far, exercise and nutrition-based interventions have shown the highest level of evidence to delay worsening^39^, although there is also evidence that multifactorial interventions including cognitive training may be beneficial^19,40,41^. This aligns well with our findings that impaired cognitive function predicts frailty worsening. Gender differences in both, transition patterns as well as transition predictors support approaches to take a gender aspect into account, especially in multifactorial frailty interventions. Studies comparing the clinical effectiveness of frailty intervention by gender, however, are still missing^39^.

The strengths of our study include a very old study population, a detailed phenotyping and its longitudinal design. This is the first time that in a high-aged German cohort prediction models for frailty transition stratified by gender were developed. Another strength is the thorough methodological approach used for developing the prediction models following the recommended TRIPOD guidelines^20^. By linking primary (BIS) and secondary (AOK claims) data, we have compiled a large, complimentary and valid health data set including date of death facilitating the imputation of missing data.

With regard to limitations, one may argue that 2.1 years of follow-up is short. However, in high-aged persons an observation of one year seemed sufficient to analyze transition effectively^12^. Although we may have missed intermediate transitions between visits, our transition rates fit in well with numbers published for up to 4 years follow-up. The 10% loss to follow-up during the observation period may have introduced selection bias. Regardless of gender, frail older adults were more often lost to follow-up. By using the claims data source, our study could utilize claims data for those participants to support the imputation of missing data for the prediction models. Therefore, we consider our models, based on a solid and well-defined dataset, as reliable. Secondly, we did not use the identical exclusion criteria as Fried, who excluded conditions which potentially present frailty characteristics as a consequence of a single disease, e.g. Parkinson^2^. This would have applied to only 26 individuals (2.2%) in our study. However, the aim of this study was not to report a representative prevalence for frailty but to describe gender-specific frailty transition and identify predictors. Since we investigated the dynamic of frailty transition using the same instrument over time, we assume our deviation from the Fried criteria to be negligible. Thirdly, with our methodological approach, models for the prediction of no change in frailty could not be developed, probably because many of the factors predict both worsening and improvement but in opposite directions. Lastly, limited by our transition numbers we were not able to include all a priori identified knowledge-based predictors in the modelling. It can be postulated that some of them might improve the prediction models especially for men. Furthermore, few variables identified in the bootstrap selection process did not show statistical significance when finally applied in the original imputed data set, presumably because they are weaker or less frequent predictors. Before potentially implementing the models in practice they require external validation.

In conclusion, our study demonstrates gender differences in the pattern of worsening and improvement of frailty as well as in predictors of transition: Women, initially more frequently frail, experience less worsening and more improvement compared to men. Women and men have different predictors of worsening. Thus, these newly developed prediction models may trigger further clinical investigation and model evaluation.

## Supplementary Information

Supplementary Information.

## Data Availability

There are no linked research data sets for this study. The data are available from the corresponding author OR from the study PI upon reasonable request.
