# Supplementary material for: Gender differences in frailty transition and its prediction in community-dwelling old adults
Source: Sci Rep. 2022 May 5;12:7341. doi: 10.1038/s41598-022-11358-7 (PMC9072330; doi:10.1038/s41598-022-11358-7)
Supplement: Supplementary file 1 — Supplementary Information. [file 41598_2022_11358_MOESM1_ESM.docx]

**Gender differences in frailty transition and its prediction in community-dwelling old adults**

Nina Mielke^1^*, Alice Schneider^1,2^, Dörte Huscher^1,2^, Natalie Ebert^1^, Elke Schäffner^1^

^1^ Charité –Universitätsmedizin Berlin, corporate member of Freie Universität Berlin, Humboldt-Universität zu Berlin, and Berlin Institute of Health, Institute of Public Health, Berlin, Germany

^2^ Charité –Universitätsmedizin Berlin, corporate member of Freie Universität Berlin, Humboldt-Universität zu Berlin, and Berlin Institute of Health, Institute of Biometry and Clinical Epidemiology, Berlin, Germany

**Appendix**

1. **Supplement A:** Frailty Assessment
2. **Supplement Table 1:** Frailty transition after 2.1 years of Follow-Up by gender, n (%).
3. **Supplement Table 2:** Summary of missing values, n (%)
4. **Supplement Table 3:** Comparison of the characteristics of participants with any missing value and those with complete data
5. **Supplement Figure 1:** Calibration plots of the prediction models for improvement and for worsening by gender
6. **Supplement A**

**Frailty Assessment**

Frailty status was determined by a modified Frailty score according to Fried [1]. Frailty was defined as having at least three of the following five items: Shrinking (1) was defined as unintentional weight loss of ≥ 5% in the past year; exhaustion (2) was assessed using two questions of the German version of the Centers for Epidemiologic Studies Depression scale [2]: How often in the last week did you feel this way?” (a) I felt that everything I did was an effort; (b) I could not get going. Participants answering a moderate amount of the time (3–4 days) or more frequently to either of these questions were categorized as frail. Low physical activity (3) was defined as answering “less than once a week” to the question “How often a week are you physically active for more than 30 minutes? (at least 30 minutes of brisk walking)”. Slowness (4) was assessed by using the Timed Up and Go test [3]. Participants were classified as slow if they needed 15 sec or more for the distance [4]. Weakness (5) was assessed by using the highest of six measurements (three measurements on each hand) with a dynamometer (Smedley, Scandidact) in kilograms. Low hand grip strength was then stratified by BMI and gender using population independent cut-off values [1]. Participants with one or two of the five items were defined as prefrail and participants with none of the five items were defined as robust.

**Frailty Assessment - Replacement of missing values**

If slowness or weakness were not determinable, it was assumed that this was due to the lack of feasibility of the participants and the person was then categorized as frail for this item. This was true for slowness (frailty baseline: 146 (13%); follow up: 151 (17%)) and for weakness (frailty baseline: 66 (6%); follow up: 59 (6%)). If BMI was missing for classification of participants to be frail in the frailty item weakness, the classification was stratified by gender only (frailty baseline: 17 (1%); follow up: 9 (1%)) [5]. If it was not possible to determine the weight loss in the past year due to missing measurement of body weight in the visit before frailty baseline, we used the body weight from another previous study visit (frailty baseline: 30 (3%); follow up: 8 (1%)). The relative weight loss per year was calculated to classify a participant as frail in terms of weight loss.

If there were missing values in addition, these were also replaced if possible. This was the case 1) if there was only one missing in the five items and the participant was frail in another item, the participant was defined as prefrail (this applied to 3 participants for each visit) and 2) if the participant was frail in at least three of the five items and any missing values in the other two items, the participant was defined as frail (at frailty baseline this applied to 28 participants, at follow up to 14 participants).

**References**

1. Fried LP, Tangen CM, Walston J, et al.; Frailty in Older Adults: Evidence for a Phenotype. *The Journals of Gerontology Series A: Biological Sciences and Medical Sciences* 2001;**56**(3):M146-M157. doi: 10.1093/gerona/56.3.M146.

2. Messinstrumente und Testverfahren in der Schmerzmedizin. *Schmerzdokumentation in der Praxis: Klassifikation, Stadieneinteilung, Schmerzfragebögen*. Berlin, Heidelberg: Springer Berlin Heidelberg, 2005, 49-118.

3. Podsiadlo D, Richardson S; The timed "Up & Go": a test of basic functional mobility for frail elderly persons. *J Am Geriatr Soc* 1991;**39**(2):142-8.

4. Savva GM, Donoghue OA, Horgan F, et al.; Using timed up-and-go to identify frail members of the older population. *J Gerontol A Biol Sci Med Sci* 2013;**68**(4):441-6. doi: 10.1093/gerona/gls190.

5. Lauretani F, Russo CR, Bandinelli S, et al.; Age-associated changes in skeletal muscles and their effect on mobility: an operational diagnosis of sarcopenia. *J Appl Physiol (1985)* 2003;**95**(5):1851-60. doi: 10.1152/japplphysiol.00246.2003.

**2) Supplement Table 1: Frailty transition after 2.1 years of Follow-Up by gender, n (%).** Numbers in the left column indicate the number of participants with respective frailty status at baseline, and column percentages sum to 100% in each stratum. The columns to the right indicate the frailty status at follow-up and the percentages are row percentages.

|  |  |  | |  | | Frailty status at Follow-up | | | | | |
| --- | --- | --- | --- | --- | --- | --- | --- | --- | --- | --- | --- |
|  |  |  |  | | Robust | | Prefrail | Frail | Deceased | Loss to FU | Missings in Frailty status |
| Frailty status at  Baseline | Total |  | 1158 (100) | | 197 (17) | | 423 (37) | 276 (24) | 133 (12) | 113 (10) | 16 (1) |
|  |  | Robust | 225 (19) | | 107 (48) | | 84 (37) | 9 (4) | 5 (2) | 13 (6) | 7 (3) |
|  |  | Prefrail | 532 (46) | | 84 (16) | | 269 (51) | 96 (18) | 32 (6) | 47 (9) | 4 (0.8) |
|  |  | Frail | 401 (35) | | 6 (2) | | 70 (18) | 171 (43) | 96 (24) | 53 (13) | 5 (1) |
|  |  |  |  | |  | |  |  |  |  |  |
|  | Women |  | 637 (100) | | 106 (17) | | 237 (37) | 164 (26) | 60 (9) | 59 (9) | 11 (2) |
|  |  | Robust | 115 (18) | | 52 (45) | | 48 (42) | 5 (4) | 2 (2) | 4 (4) | 4 (4) |
|  |  | Prefrail | 282 (44) | | 48 (17) | | 142 (50) | 49 (17) | 15 (5) | 25 (9) | 3 (1) |
|  |  | Frail | 240 (38) | | 6 (3) | | 47 (20) | 110 (46) | 43 (18) | 30 (13) | 4 (2) |
|  |  |  |  | |  | |  |  |  |  |  |
|  | Men |  | 521 (100) | | 91 (18) | | 186 (36) | 112 (22) | 73 (14) | 54 (10) | 5 (1) |
|  |  | Robust | 110 (21) | | 55 (50) | | 36 (33) | 4 (4) | 3 (3) | 9 (8) | 3 (3) |
|  |  | Prefrail | 250 (48) | | 36 (14) | | 127 (51) | 47 (19) | 17 (7) | 22 (9) | 1 (0.4) |
|  |  | Frail | 161 (31) | | 0 (0) | | 23 (14) | 61 (38) | 53 (33) | 23 (14) | 1 (0.6) |

**3) Supplement Table 2: Summary of missing values, n (%)**

|  | **Total**  **n = 1158** | | **Women**  **N = 637** | | **Men**  **N = 521** |
| --- | --- | --- | --- | --- | --- |
| **Participants with any missing value** | 248 (21) | | 141 (22) | | 107 (21) |
| Participants with  1 missing value  2 missing value  3 missing value  4 missing value  5 missing value  6 missing value | 183 (16)  27 (2)  13 (1)  15 (1)  5 (0.4)  5 (0.4) | | 103 (16)  18 (3)  7 (1)  8 (1)  3 (0.5)  2 (0.3) | | 80 (15)  9 (2)  6 (1)  7 (1)  2 (0.4)  3 (0.6) |
| **Number of missing values per predictor and outcome*** | |  | |  | |
| Education (CASMIN-short) | 5 (0.4) | | 1 (0.2) | | 4 (0.8) |
| Smoking | 4 (0.3) | | 3 (0.5) | | 1 (0.2) |
| Self-rated health | 3 (0.3) | | 2 (0.3) | | 1 (0.2) |
| Living alone | 38 (3) | | 25 (4) | | 13 (2) |
| BMI in kg/m^2^ | 17 (1) | | 7 (1) | | 10 (2) |
| Polypharmacy | 1 (0.1) | | 0 (0) | | 1 (0.2) |
| Cognitive impairment | 68 (6) | | 32 (5) | | 36 (7) |
| Diabetes mellitus | 15 (1) | | 8 (1) | | 7 (1) |
| eGFR_BIS2_ < 60 ml/min/1.73m² | 34 (3) | | 22 (3) | | 12 (2) |
| ACR ≥ 30 mg/g | 77 (7) | | 49 (8) | | 28 (5) |
| Frailty transition | 129 (11) | | 70 (11) | | 59 (11) |

*no missing values for the predictors: age, frailty status at baseline, heart failure, stroke, cancer, osteoarthritis, hospitalisation, COPD; BMI: body mass index; eGFR: estimated glomerular filtration rate; ACR: albumin to creatinine ratio; COPD: chronic obstructive lung disease

**4) Supplement Table 3: Comparison of the characteristics of participants with any missing value and those with complete data**

|  |  |  |  | **Women** |  |  |  |  |  | **Men** |  |  |
| --- | --- | --- | --- | --- | --- | --- | --- | --- | --- | --- | --- | --- |
|  |  | **Total** |  | **With missings** |  | **Complete data** |  | **Total** |  | **With missings** |  | **Complete data** |
| n |  | 637 |  | 141 |  | 496 |  | 521 |  | 107 |  | 414 |
| Age in years, mean (SD) |  | 84.1 (5.6) |  | 85.7 (5.9) |  | 83.6 (5.4) |  | 84.8 (5.7) |  | 85.4 (6.3) |  | 84.6 (5.5) |
| Education (CASMIN-short), n (%) |  |  |  |  |  |  |  |  |  |  |  |  |
| Low |  | 417 (65) |  | 94 (67) |  | 323 (65) |  | 269 (52) |  | 54 (50) |  | 215 (52) |
| Middle |  | 148 (23) |  | 33 (23) |  | 115 (23) |  | 87 (17) |  | 18 (17) |  | 69 (17) |
| High  unknown |  | 71 (11) 1 (0.2) |  | 13 (9) 1 (1) |  | 58 (12) n.a. |  | 161 (31)  4 (1) |  | 31 (29) 4 (4) |  | 130 (31) n.a. |
| Smoking, n (%)  never  ever  unknown |  | 458 (72)  176 (28)  3 (0.5) |  | 109 (77)  29 (21)  3 (2) |  | 349 (70)  147 (30)  n.a. |  | 164 (31)  356 (68)  1 (0.2) |  | 35 (33)  71 (66)  1 (1) |  | 129 (31)  285 (69)  n.a. |
| Self-rated health, n (%) |  |  |  |  |  |  |  |  |  |  |  |  |
| excellent / good |  | 267 (42) |  | 49 (35) |  | 218 (44) |  | 254 (49) |  | 44 (41) |  | 210 (51) |
| moderate / poor / very poor  unknown |  | 368 (58)  2 (0.3) |  | 90 (64)  2 (1) |  | 278 (56)  n.a. |  | 266 (51)  1 (0.2) |  | 62 (58)  1 (1) |  | 204 (49)  n.a. |
| Living alone, n (%)  unknown |  | 393 (62)  25 (4) |  | 75 (53)  25 (18) |  | 318 (64)  n.a. |  | 159 (31)  13 (2) |  | 33 (31)  13 (12) |  | 226 (30)  n.a. |
| BMI in kg/m^2^, n (%)  <25  25-29.9  ≥ 30  unknown |  | 225 (35)  251 (39)  154 (24)  7 (1) |  | 54 (38)  47 (33)  33 (23)  7 (5) |  | 171 (34)  204 (41)  121 (24)  n.a. |  | 152 (29)  258 (50)  101 (19)  10 (2) |  | 34 (32)  43 (40)  20 (19)  10 (9) |  | 118 (29)  215 (52)  81 (20)  n.a. |
| Polypharmacy, n (%)  unknown |  | 470 (74)  0 (0) |  | 103 (73)  0 (0) |  | 367 (74)  n.a. |  | 378 (73)  1 (0.2) |  | 80 (75)  1 (1) |  | 298 (72)  n.a. |
| Hospitalization, n (%)  0  1-2  ≥3 |  | 284 (45)  230 (36)  123 (19) |  | 59 (42)  48 (34)  34 (24) |  | 225 (45)  182 (37)  89 (18) |  | 205 (39)  218 (42)  98 (19) |  | 41 (38)  49 (46)  17 (16) |  | 164 (40)  169 (41)  81 (20) |
| Cognitive impairment, n (%)  unknown |  | 55 (9)  32 (5) |  | 22 (16)  32 (23) |  | 33 (7)  n.a. |  | 40 (8)  36 (7) |  | 19 (18)  36 (34) |  | 21 (5)  n.a. |
| Stroke, n (%) |  | 75 (12) |  | 18 (13) |  | 57 (11) |  | 81 (16) |  | 26 (24) |  | 55 (13) |
| Congestive Heart failure, n (%) |  | 301 (47) |  | 82 (58) |  | 219 (44) |  | 250 (48) |  | 56 (52) |  | 194 (47) |
| Diabetes mellitus, n (%)  Unknown |  | 171 (27)  8 (1) |  | 43 (30)  8 (6) |  | 128 (26)  n.a. |  | 170 (33)  7 (1) |  | 42 (39)  7 (7) |  | 128 (31)  n.a. |
| Cancer, n (%) |  | 134 (21) |  | 34 (24) |  | 100 (20) |  | 181 (35) |  | 31 (29) |  | 150 (36) |
| Osteoarthritis, n (%) |  | 558 (88) |  | 123 (87) |  | 435 (88) |  | 377 (72) |  | 78 (73) |  | 299 (72) |
| COPD, n (%) |  | 254 (40) |  | 64 (45) |  | 190 (38) |  | 196 (38) |  | 51 (48) |  | 145 (35) |
| eGFR_BIS2_ < 60 ml/min/1.73m^2^, n (%)  unknown |  | 468 (73)  22 (3) |  | 96 (68)  22 (16) |  | 372 (75)  n.a. |  | 379 (73)  12 (2) |  | 74 (69)  12 (11) |  | 305 (74)  n.a. |
| ACR ≥30 in mg/g  unknown |  | 137 (22)  49 (8) |  | 25 (18)  49 (35) |  | 112 (23)  n.a. |  | 169 (32)  28 (5) |  | 29 (27)  28 (26) |  | 140 (34)  n.a. |
| Frailty baseline status, n (%) |  |  |  |  |  |  |  |  |  |  |  |  |
| robust |  | 115 (18) |  | 14 (10) |  | 101 (20) |  | 110 (21) |  | 17 (16) |  | 93 (22) |
| prefrail  frail |  | 282 (44)  240 (38) |  | 44 (31)  83 (59) |  | 238 (48)  157 (32) |  | 250 (48)  161 (31) |  | 41 (38)  49 (46) |  | 209 (50)  112 (27) |
| Frailty transition after 2.1 years  improvement  no change  worsening  unknown |  | 101 (16)  304 (48)  162 (25)  70 (11) |  | 13 (10)  36 (26)  22 (16)  70 (50) |  | 88 (18)  268 (54)  140 (28)  n.a. |  | 59 (11)  243 (47)  160 (31)  59 (11) |  | 3 (3)  18 (17)  27 (25)  59 (55) |  | 56 (14)  225 (54)  133 (32)  n.a. |

n.a. (not applicable); variables without any missing value: age, living alone, congestive heart failure, cancer, osteoarthritis, COPD, number of hospitalization, stroke, SD: standard deviation; BMI: body mass index; COPD: chronic obstructive lung disease; eGFR: estimated glomerular filtration rate; ACR: albumin to creatinine ratio

**5) Calibration Plots**


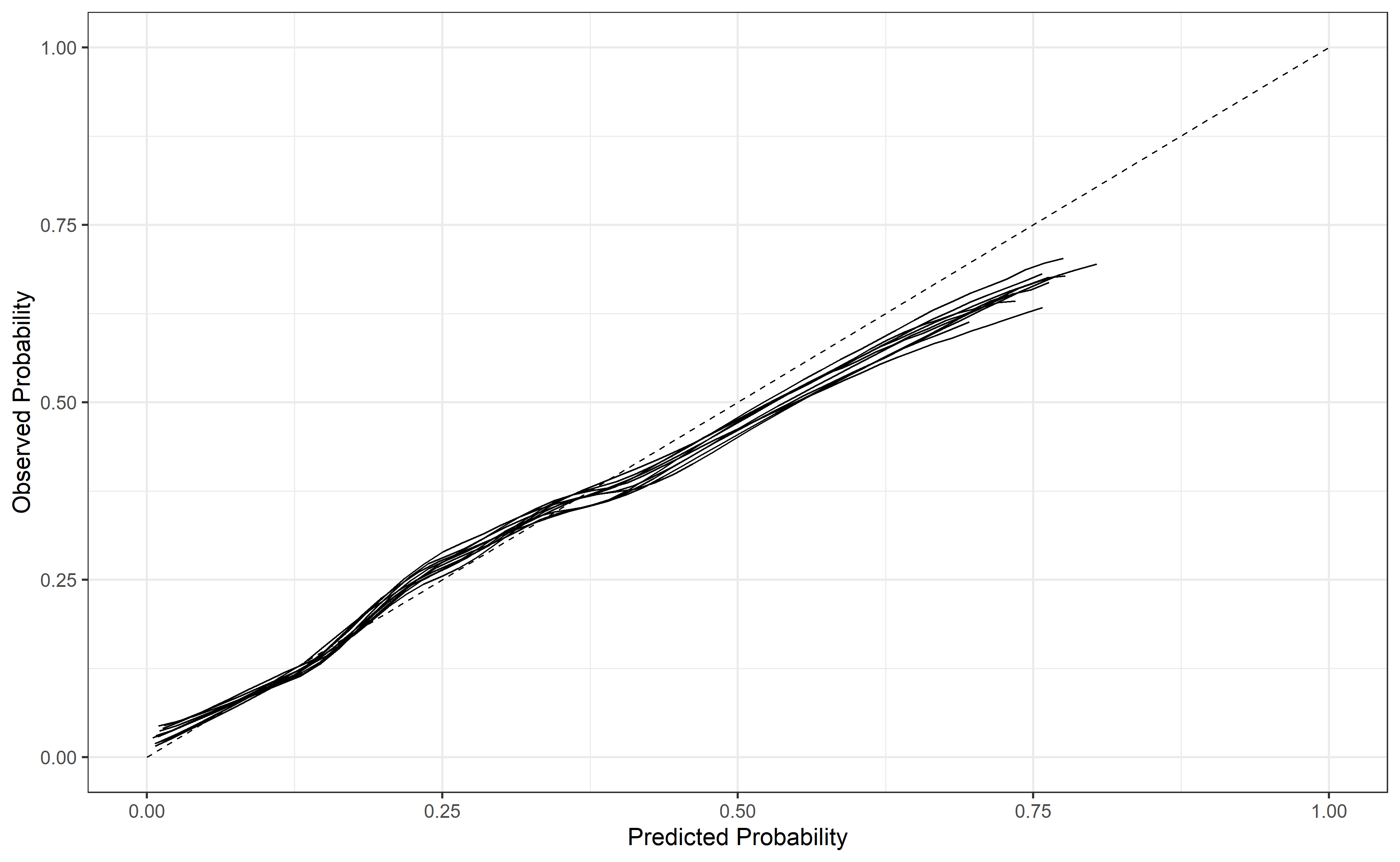


**Figure 1A:** Calibration plot for improvement in women. Each line represents the calibration for one imputed data set, the dotted line represents a perfect calibration. The calibration intercept ranged from -0.16 to -0.09 and the calibration slope from 0.84 – 0.90.


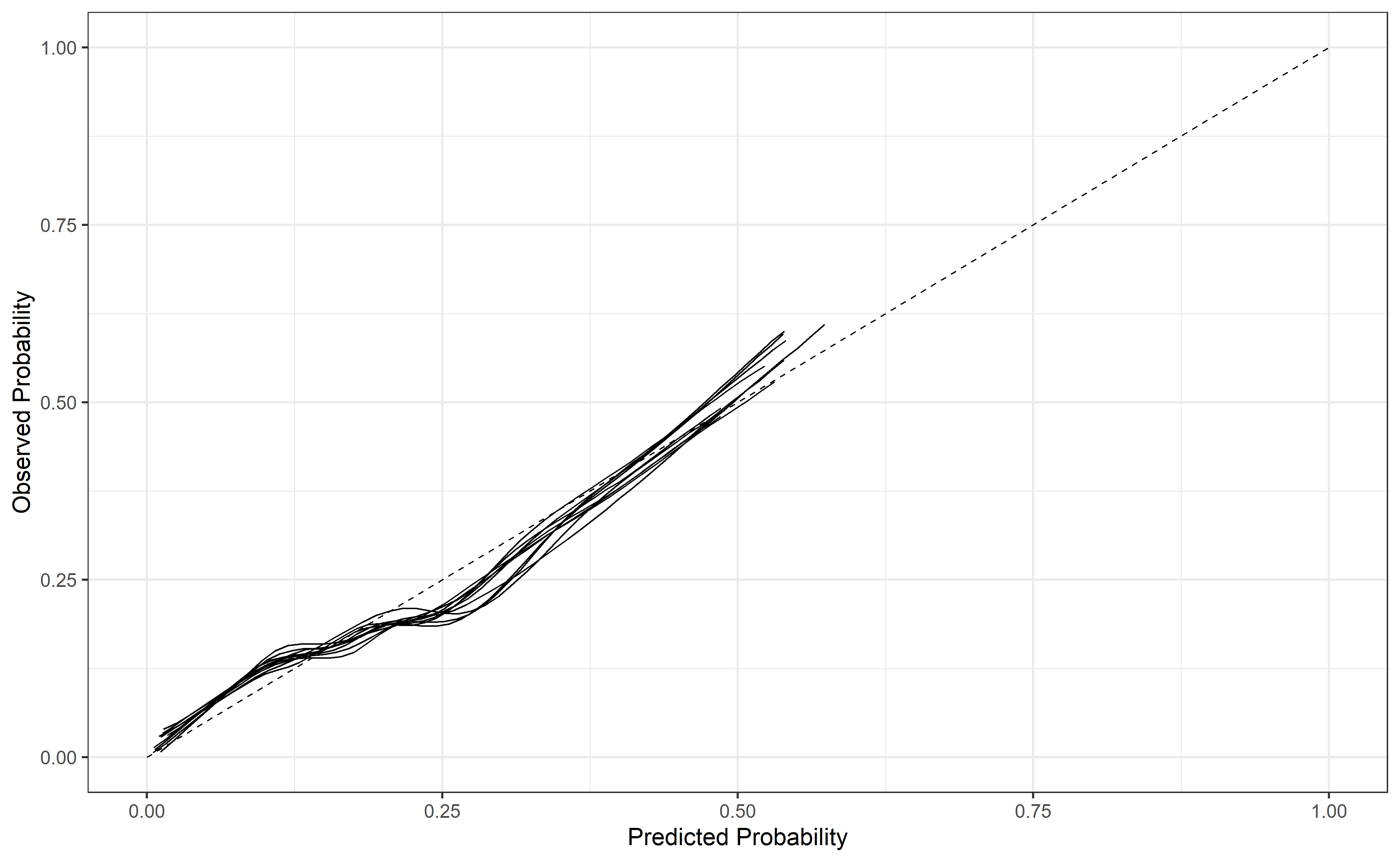


**Figure 1B:** Calibration plot for improvement in men. Each line represents the calibration for one imputed data set, the dotted line represents a perfect calibration. The calibration intercept ranged from -0.32 to -0.21 and the calibration slope from 0.77 – 0.84.


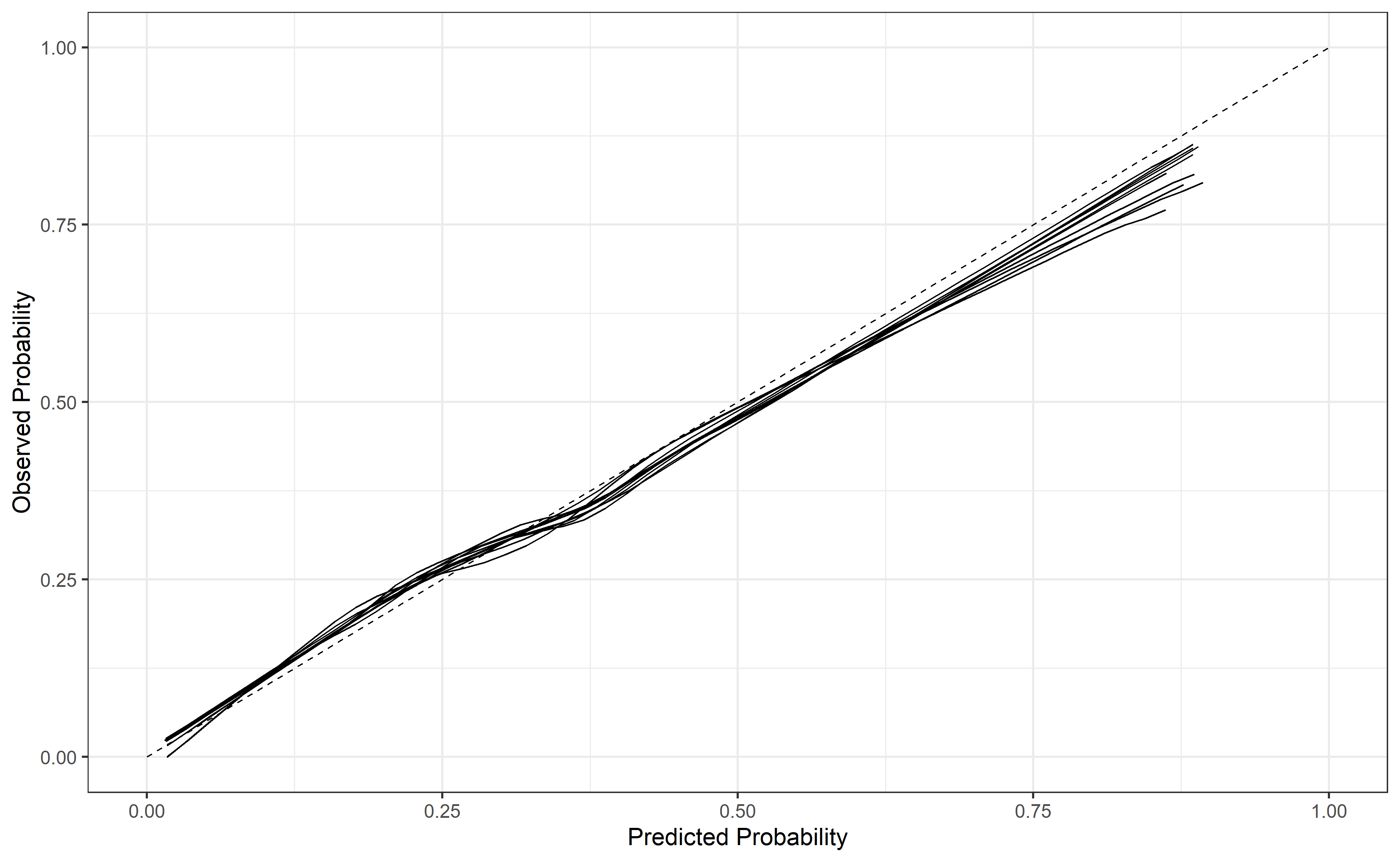


**Figure 1C:** Calibration plot for worsening in women. Each line represents the calibration for one imputed data set, the dotted line represents a perfect calibration. The calibration intercept ranged from -0.09 to -0.07 and the calibration slope from 0.88 – 0.90.


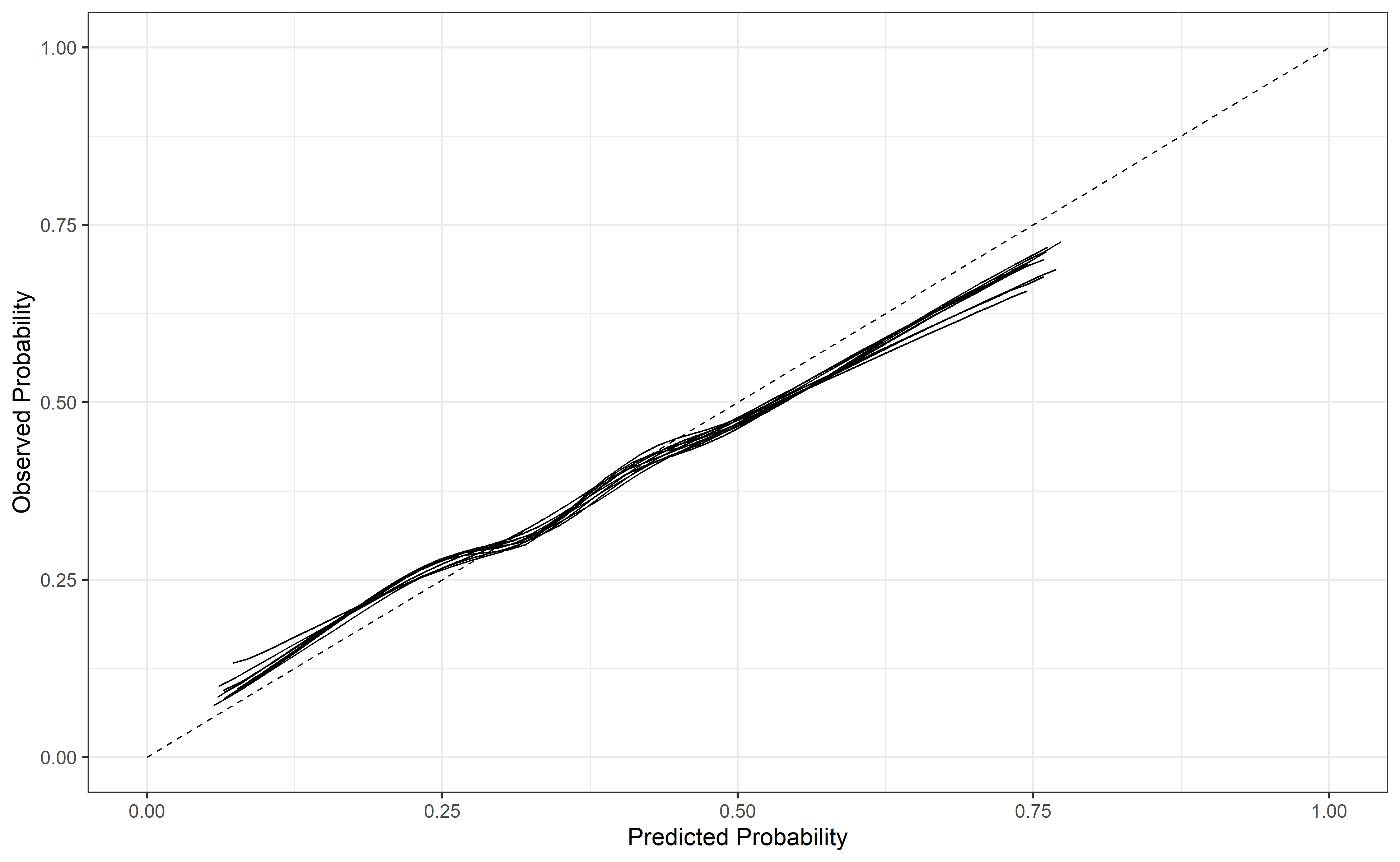


**Figure 1D:** Calibration plot for worsening in men. Each line represents the calibration for one imputed data set, the dotted line represents a perfect calibration. The calibration intercept ranged from -0.09 to -0.07 and the calibration slope from 0.84 – 0.88.
